# Pharmacist-led teaching as a longitudinal theme for medical school curriculums - a solution for reducing prescribing errors in junior doctors?

**DOI:** 10.1186/s12909-019-1632-9

**Published:** 2019-05-29

**Authors:** Naomi Lloyd

**Affiliations:** School of Medicine, University of Liverpool, Merseyside, UK

**Keywords:** Junior, Doctor, Prescribing, Errors, Pharmacist, Structured, Teaching

## Abstract

Medication errors are a significant problem faced by health services internationally.

Prescribing errors are a common preventable source of morbidity and mortality, and as such it is our duty as healthcare professionals to minimise them as much as possible.

Many prescriptions, and errors, are written by junior doctors. Which raises the question of whether the medical school curriculum could be changed to better prepare students for prescribing.

There is a great deal of evidence in the literature describing how pharmacist-led teaching in later years of medical school has a beneficial effect on the quality of prescriptions written by junior doctors. In addition, this style of teaching leads to a reduction in the number of prescribing errors that occur. However, many of these papers still reported that students were still apprehensive about their prescriptions after the teaching programmes, and a number of their prescriptions still contained some inaccuracies.

The University of Liverpool organises ‘Safe Prescribing’ teaching sessions during years 3–5 of the undergraduate medical curriculum. This programme consistently receives positive feedback and results in students feeling more comfortable and confident in a variety of prescribing scenarios.

Incorporating pharmacist-led teaching as a longitudinal theme from the early stages of medical school is one way positive habits can be built and reinforced. This may in turn reduce the number of prescribing errors that occur, both in junior doctors and more senior doctors as these clinicians progress through their training armed with an effective skill set.

## Background

I read with interest ‘A pilot study of a pharmacist-led prescribing program for final-year medical students’ [1]. As a fourth year medical student at The University of Liverpool, I have been receiving regular pharmacist-led teaching since my third year, and would like to contribute to the key ideas discussed.

As this paper details, medication errors are a significant problem faced by health services internationally. Prescribing errors are a common preventable source of morbidity and mortality, and as such it is our duty as healthcare professionals to minimise them as much as possible. As a considerable amount of prescriptions, and errors, are written by junior doctors, this raises the question of whether medical school curriculums could better prepare students for prescribing [1]. A systematic review by Brinkman et al. found final year medical students lacked a number of competencies. Emphasising that to combat this, changes need to be made to the undergraduate medical curriculum [2].

## Main text

Newby et al. described how an 8 week pharmacist-led programme led to dramatic improvements in the quality of prescribing; an increase in the amount of appropriate prescriptions, and a reduction in the number of inaccurate and potentially harmful prescriptions. However the number of inappropriate but not harmful prescriptions increased, and there was still an equally high number of students prescribing appropriately but apprehensively before and after the programme [1].

Recognising this shortfall, The University of Liverpool organises ‘Safe Prescribing Teaching’ in years 3–5 of the undergraduate curriculum. These are weekly teaching sessions led by hospital pharmacists during our 8 week long medicine placements. They encompass a number of topics including taking an accurate medication history and the prescribing of key medications such as antibiotics, insulins and opioids. Also incorporating frequent practice writing prescriptions. These sessions consistently receive positive feedback, with many students saying they feel remarkably more confident and comfortable in prescribing situations after the teaching.

This idea of incorporating this style of teaching earlier in the medical curriculum is supported by a paper written by Ward and Miloszewski. They demonstrated that a series of pharmacist-led tutorials led to a dramatic improvement in students’ knowledge, and were well praised by participants [3]. In addition, a study looking at the causes and consequences of inpatient prescribing errors stated “Alternative strategies for involving pharmacists should also be considered” [4]. Involving pharmacists in this style of teaching also helps to further interprofessional links and understanding, as Newby et al. found [1]. A further systematic review by Kamarudin et al. found a number of pharmacist-led programmes which resulted in a number of positive outcomes [5].

## Conclusion

By incorporating consistent and regular pharmacist-led teaching throughout medical school, rather than solely in students’ final year, good practice would be encouraged and ingrained from an early stage. If pharmacist led teaching was established as a longitudinal theme, revisited and built on regularly, positive habits would develop. Through the reinforcement of regular review, I believe this would have a substantial impact on the prescribing competencies of junior doctors.

## Data Availability

Not applicable.
